# Chemotherapy and echocardiographic indices in patients with non-Hodgkin lymphoma: the ONCO-ECHO study

**DOI:** 10.1007/s12032-017-1075-2

**Published:** 2017-12-22

**Authors:** Katarzyna Mizia-Stec, Marek Elżbieciak, Maciej T. Wybraniec, Monika Różewicz, Artur Bodys, Wojciech Braksator, Zbigniew Gąsior, Piotr Gościniak, Tomasz Hryniewiecki, Jarosław Kasprzak, Andrzej Wojtarowicz, Barbara Zdziarska, Edyta Płońska-Gościniak

**Affiliations:** 1 First Department of Cardiology, School of Medicine in Katowice, Medical University of Silesia, 47 Ziołowa St., 40-635 Katowice, Poland; 2 Department of Congenital Cardiac Defects, The Cardinal Stefan Wyszynski Institute of Cardiology, Warsaw, Poland; 3 Stefan Cardinal Wyszynski Regional Hospital, Lublin, Poland; 4 Department of Cardiology, Hypertension and Internal Medicine, 2nd Medical Faculty, Medical University of Warsaw, Warsaw, Poland; 5 Second Department of Cardiology, School of Medicine in Katowice, Medical University of Silesia, Katowice, Poland; 6 Department of Cardiology, Province Hospital, Szczecin, Poland; 7 Department of Heart Valve Diseases, The Cardinal Stefan Wyszynski Institute of Cardiology, Warsaw, Poland; 8 Department of Cardiology, Medical University of Lodz, Lodz, Poland; 9 Department of Cardiology, Pomeranian Medical University, Szczecin, Poland; 10 Department of Hematology, Pomeranian Medical University, Szczecin, Poland

**Keywords:** NHL, Non-Hodgkin lymphoma, Chemotherapy, Cardiotoxicity

## Abstract

The cardiotoxicity of chemotherapy (CTx) for non-Hodgkin’s lymphomas is not well recognized. In order to facilitate individual risk counseling for patients, we analyzed the effect of CTx on echocardiographic indices in regard to clinical data in patients treated for non-Hodgkin’s lymphoma (NHL). A prospective multicenter ONCO-ECHO trial included 67 patients with NHL (45 patients with DLBCL (diffuse large B cell lymphoma) and 22 with non-DLBCL). Patients received standard CTx, primarily R-CHOP, CHOP, R-COP and COP regimens. Clinical data and echocardiographic indices were obtained at baseline, 3-, 6- and 12-month follow-up. The primary end point representing CTx cardiotoxicity was defined as *a* ≥ 10% decrease in the left ventricular ejection fraction (LVEF) during 12-month observation. In a 12-month follow-up five (7.5%) deaths occurred, while no clinical manifestations of heart failure were reported. There was an increase in left ventricular end-systolic diameter (*p* = 0.002) and *E*/*e*′ index (*p* = 0.036) in 12-month observation. Preexisting coronary artery disease was associated with significant decrease in the ΔLVEF (*p* = 0.008), increase in ΔLVEDV (*p* = 0.03) and ΔLVESV (*p* = 0.02) and increase in the Δ left atrium diameter (*p* = 0.02); while history of arterial hypertension was related to significant decrease in the ΔLVEF (*p* = 0.039), diabetes mellitus was related to significant increase in the Δ*E*/*e*′ index (*p* = 0.002). The primary end point was reported in ten (14.9%) patients. There were no independent risk factors for cardiotoxicity in the study population. Chemotherapy administered to NHL patients may induce dilatation and impaired LV diastolic function. Standard cardiovascular risk factors may predispose patients to negative LV remodeling.

## Introduction

The ever-growing incidence of neoplastic disease necessitates the wide application of chemotherapeutic agents. The management of adverse events following the use of chemotherapy (CTx) regimens has become one of the mainstays of contemporary complex oncological care [1]. CTx-related complications frequently pertain to the cardiovascular system [2]. There is a great deal of scientific evidence concerning the evident cardiotoxicity of anthracyclines, alkylating antineoplastic agents and platinum analogs [2–4]. Cardiotoxicity can be associated with both clinically symptomatic cardiovascular dysfunction secondary to CTx [3] and subclinical cardiac damage, which might have potential prognostic implications [4]. In both instances, close cardiovascular supervision is of the utmost importance [5]. It is noteworthy that there is paucity of data in the literature concerning the contemporary imaging of early cardiac abnormalities secondary to CTx-related cardiotoxicity [6]. Based on this premise, a multicenter ONCO-ECHO study was established [7], which comprised an echocardiographic assessment of cardiotoxicity in different forms of malignant neoplasms, including non-Hodgkin’s lymphomas [7]. Non-Hodgkin’s lymphomas (NHL) constitute a heterogeneous group of neoplasms that derive from lymphocytes and their precursor cells, or from cells that originate from transformed B or T lymphocytes or histiocytes. The vast majority of lymphomas stem from B line lymphocytes (86%), whereas the rest derives from T line lymphocytes (12%) or NK cells (2%) [8]. NHL belongs to one of the most prevalent neoplasms in Poland and worldwide. In 2014, 3328 new cases of NHL and 1814 NHL-related deaths were reported in Poland [9].

The management of NHL is based on CTx, which is fraught with many adverse actions, including those related to cardiovascular system, widely known as cardiotoxicity [2–4, 10, 11]. Examples of contemporary regimens of CTx that are applied for NHL are: R-COP (rituximab, cyclophosphamide, vincristine, prednisone); R-CHOP (additionally doxorubicin); DHAP (cisplatin, dexamethasone, cytarabine); R-ICE (rituximab, ifosfamide, carboplatin, etoposide); hyper-CVAD/HDMTX-Ara-C (cyclophosphamide, doxorubicin, vincristine, dexamethasone/methotrexate, folinic acid, cytarabine); RFC (rituximab, fludarabine, cyclophosphamide); R-FCM (fludarabine, cyclophosphamide, mitoxantrone); cladribine; R-B (rituximab, bendamustine) [8, 12].

One of the crucial elements of different chemotherapeutic regimens in patients with NHL is anthracyclines (doxorubicin, daunorubicin, epirubicin, mitoxantrone), which presumably inflict considerable injury on cardiomyocytes [13, 14]. In different populations of oncological patients, several risk factors for anthracycline-induced cardiomyopathy have been determined, such as the cumulative dose of anthracyclines, prior or planned thoracic radiation therapy, age > 65 years old, coexisting cardiovascular disease, diabetes mellitus and the simultaneous use of other cardiotoxic drugs (trastuzumab, cyclophosphamide, fluorouracil) [2, 4, 15]. Insufficient data regarding the cardiac adverse actions of CTx regimens designed for NHL treatment and their possible risk factors exist in the literature. Accordingly, the study aimed to investigate the impact of CTx on echocardiographic indices and clinical course of the disease in patients diagnosed with NHL.

## Materials and methods

This prospective, multicenter ONCO-ECHO study comprised 67 adult patients with the diagnosis of NHL (30 men, 44.8%) at a median age of 58 years (51, 68). The study was conducted in 8 tertiary reference cardiovascular medical centers in Poland between 2013 and 2015 [7]. All the institutions cooperated with local departments of oncology. The study was approved by Ethics Committee of the Pomeranian Medical University in Szczecin. All the study participants granted written informed consent for enrollment in the study. The inclusion criteria involved the diagnosis of non-Hodgkin lymphoma according to universal 2012 National Comprehensive Cancer Network (NCCN) criteria for the diagnosis of NHL [8]. The exclusion criteria entailed: age < 18 years old, baseline left ventricular ejection fraction (LVEF) < 55%, regional wall motion abnormalities at rest, myocardial hypertrophy (any LV segment ≥ 13 mm), moderate or severe valvular heart disease, prior history of CTx or radiotherapy, lack of written informed consent and inadequate acoustic window [7].

All patients received standard CTx—data presented in Table 1. A compound of demographic and clinical variables (past medical history, New York Heart Association symptomatic class, heart rate, blood pressure, etc.), and potential indices of cardiotoxicity (B-type natriuretic peptide (BNP) and troponin T concentration), as well as echocardiographic parameters, were assessed. Clinical and echocardiographic checkup was performed on initial admission prior to CTx initiation and 3, 6 and 12 months following enrollment. Death, heart failure and unplanned cardiovascular hospitalization during the 12-month follow-up were analyzed.

**Table 1:** Demographic and clinical characteristics of the study population

Variable	*N* = 67 (100%)
Male	30 (44.8%)
DLBCL	45 (67.2%)
Non-DLBCL	22 (32.8%)
CTx regimen	
R-CHOP	44 (66%)
CHOP	9 (13%)
R-COP	3 (4.5%)
COP	3 (4.5%)
Other regimens	8 (12%)
Anthracycline—in total	58 (86.6%)
Rituximab—in total	50 (74.6%)
Adjunctive therapy	
Radiation therapy	3 (4.5%)
Beta blockers (during CTx cycles)	15 (22.4%)
ACEI (during CTx cycles)	19 (28.4%)
Diuretics (during CTx cycles)	13 (19.4%
Baseline laboratory parameters	
Hematocrit (%)	39.1 (36.4; 42.7)
RBC (mln/mm^3^)	4.4 (3.9; 4.8)
WBC (1/mm^3^)	6.8 (4.7; 9.8)
PLT (× 1000/mm^3^)	223 (159; 276)
Serum creatinine (mg/dL)	0.82 (0.70; 0.96)
Total cholesterol (mg/dL)	210 (167; 245)
Alanine transferase (U/L)	18 (12; 24)
Lactic dehydrogenase (U/L)	215 (170; 323)
BNP (pg/mL)	246 (169; 315)
Troponin *T* (ug/mL)	0.007 (0.005; 0.009)
Comorbidities and cardiovascular risk factors	
Coronary artery disease	7 (10.5%)
Arterial hypertension	28 (41.8%)
Diabetes mellitus	9 (13.4%)
Stroke/TIA in anamnesis	2 (3%)
Cigarette smoking	30 (44.8%)
Family history of cardiovascular disease	29 (43.3%)
Mild mitral valve insufficiency at baseline	31 (46.3%)

*RBC* red blood cell count, *WBC* white blood cell count, *ACEI* angiotensin-converting enzyme inhibitors, *CTx* chemotherapy, *TIA* transient ischemic attack, *DLBCL* diffuse large B cell lymphoma, *MI* myocardial infarction, *R*-*CHOP*/*CHOP* cyclophosphamide, vincristine, prednisone, doxorubicin ± rituximab, *R*-*COP*/*COP* cyclophosphamide, vincristine, prednisone ± rituximab

The primary end point of the study/CTx-related cardiotoxicity was defined as the ≥ 10% decrease in LVEF throughout the 12 months, relative to baseline values.

Transthoracic echocardiography (TTE) was performed using Vivid 7 (GE Healthcare) or EPIQ 7 (Philips) device by experienced investigators with National Certification of experts in echocardiography fields. The following measurements were made according to American Society of Echocardiography (ASE) [16] recommendation: diameters and both systolic and diastolic function of the left ventricle; diameters of right ventricle; diameters and volume of left atrium. Tissue Doppler imaging (TDI) allowed for measurement of peak systolic annular velocity (s’) and peak early (e’) and late myocardial diastolic velocity (a’), separately for interventricular septum and lateral wall.

The temporal variability of the assessed parameters (∆) defined as maximal shift during 12-month therapy with reference to baseline value was calculated.

### Statistical analysis

Statistical analysis was performed using licensed Statistica 10.0 software (StatSoft, Poland). Continuous variables were expressed as mean and standard deviation (SD) in case of normally distributed variables or median and 25–75% percentile boundaries in case of non-normally distributed variables. The type of distribution of every variable was verified by means of Shapiro–Wilk’s test. The homogeneity of variance was evaluated using Levene’s test. Intergroup differences were verified using Student’s *T* test for normally distributed variables. Mann–Whitney *U* test was utilized in the instance of non-normally distributed parameters. Intergroup difference with regard to qualitative variables was established by means of Pearson’s Chi-squared test with Yates correction. Temporal variability of different clinical and echocardiographic variables was evaluated using Wilcoxon’s matched pairs test and Friedman’s analysis of variance or paired sample *T* test and repeated measures analysis of variance. *P* value < 0.05 was regarded as statistically significant. Univariate and multivariate analyses of the risk of ≥ 10% decrease in LVEF in 12-month observation were performed; univariate analysis comprised estimation of odds ratio (OR) with 95% confidence limit. The completeness of data exceeded 90% in each variable assessed in the model.

## Results

Precise clinical characteristics are highlighted in Table 1. Patients predominantly suffered from the most common diffuse large B cell lymphoma (DLBCL; 67.2%), the rest was diagnosed with non-DLBCL (32.8%). In the study population, NHL was predominantly in the fourth stage according to Ann Arbor classification. Five (7.5%) deaths were reported during 12-month follow-up. All deaths were related to the progression of neoplastic disease. There were not unplanned cardiovascular hospitalizations during 12-month follow-up. The summary of demographic and clinical characteristics of the study population is presented in Table 1.

### Analysis of temporal variations of major clinical parameters

Among the patients who reached 12-month follow-up, no decrease in exercise tolerance according to NYHA classification was demonstrated (Table 2). During this period, we have not reported any fluctuations of laboratory indices of cardiotoxicity, such as troponin T concentration or BNP level. Troponin T levels were in normal range in all patients at the baseline, and only mild increase was observed in some subjects during follow-up. Serum BNP levels were increased but stable in all subjects (Table 2).

**Table 2:** Temporal fluctuations of clinical and laboratory and echocardiographic parameters in the course of observation

Variable	Baseline	3 months	6 months	12 months	*P*
	Median (25–75%) or mean ± SD				
NYHA class	0.86 ± 0.85	0.86 ± 0.85	1.0 ± 0.79	1.03 ± 0.75	0.36
BNP (pg/mL)	246 (169; 315)	270 (149; 347)	270 (150; 301)	170 (123; 310)	0.21
Troponin T (μg/L)	0.007 (0.005; 0.009)	0.01 (0.009; 0.02)	0.01 (0.005; 0.04)	0.005 (0.003; 0.015)	0.32
SBP (mmHg)	127 ± 14	125 ± 13	128 ± 12	126 ± 12	0.78
DBP (mmHg)	81 ± 10	80 ± 9	80 ± 8	80 ± 8	0.89
HR (1/min)	76.8 ± 12.5	78.4 ± 11.6	77.5 ± 10.6	73.06 ± 11.8	0.43
LVEF (%)	64.7 ± 7	64.2 ± 8.0	61.8 ± 7.7	62.3 ± 10.2	0.72
LVEDD (mL)	46.8 ± 7	47.1 ± 6.7	47.2 ± 6.3	48.1 ± 6.6	0.84
LVESD (mL)	28.8 ± 6.6	29.4 ± 6.9	30 ± 7.1	31.4 ± 6.5	0.41
LVESV (mL)	35.6 ± 17	34.5 ± 14.7	35.6 ± 16.1	41.2 ± 22.6	0.39
LVEDV (mL)	100 ± 33.5	97.3 ± 31.9	95.2 ± 35	103.9 ± 35	0.59
LVSV (mL)	64.4 ± 21.6	62.4 ± 24.8	51.5 ± 29.6	62.6 ± 20.6	0.56
*e*′ (m/s)	0.08 ± 0.03	0.08 ± 0.03	0.08 ± 0.025	0.08 ± 0.03	0.98
*a*′ (m/s)	0.1 ± 0.02	0.09 ± 0.02	0.096 ± 0.02	0.09 ± 0.02	0.92
*E*/*e*′	8.5 ± 3.0	8.7 ± 3.4	9.0 ± 3.7	8.2 ± 3.7	0.59
*s*′ mean (m/s)	0.09 ± 0.02	0.09 ± 0.02	0.085 ± 0.02	0.088 ± 0.02	0.62
RV mid-cavity (mm)	27.2 ± 5.2	26.8 ± 5.3	25 ± 5.8	25.9 ± 5.7	0.13
LA (LAX) (mm)	35.4 ± 5.5	35.4 ± 5.5	37.3 ± 4.5	36.8 ± 5.7	0.46
LAA (4Ch) (cm^2^)	17.9 ± 3.3	17.7 ± 2.9	17.9 ± 3.4	17.9 ± 2.7	0.56
LA volume (mL)	52.5 ± 14.9	53.6 ± 14.2	52.6 ± 17.4	53.1 ± 13.2	0.41
IVRT (ms)	101.6 ± 25.6	95.7 ± 25.1	71.2 ± 47.6	94.5 ± 23.4	0.29

*BNP* B-type natriuretic peptide, *SBP/DBP* systolic and diastolic blood pressure, *HR* heart rate, *LVEF* left ventricular ejection fraction, *LVEDD*/*LVESD* left ventricular end-diastolic or end-systolic diameter, *LVEDV/LVESV* left ventricular end-diastolic or end-systolic volume, *LVSV* left ventricular stroke volume, *LA* (*LAX*) left atrial diameter in parasternal long axis view, *LAA* (*4Ch*) left atrial area in four-chamber view, *LA volume* left atrial volume calculated using Simpson method, *IVRT* isovolumic relaxation time

### Analysis of temporal variations of echocardiographic indices in the whole population

None of the assessed echocardiographic parameters varied significantly in the course of 12-month follow-up (Table 2). Only change in LVESD between baseline and 12-month follow-up values (28.8 ± 6.6 vs. 31.4 ± 6.5 mm, *p* = 0.002) and change in *E*/*e*′ between baseline and 6-month follow-up (7.63 ± 2.9 vs. 8.11 ± 3.7, *p* = 0.036) reached statistical significance.

### Cardiovascular risk factors and change in echocardiographic indices

Sub-analysis denoted that patients with the diagnosis of coronary artery disease had greater decrease in LVEF (∆LVEF: − 14 vs. − 1.9%; *p* = 0.01), more pronounced increase in left ventricular end-diastolic volume (∆LVEDV: 6.0 vs. 1.6 mL, *p* = 0.03) and left ventricular end-systolic volume (∆LVESV: 29.0 vs. 2.7 mL, *p* = 0.02) and left atrial diameter in parasternal long axis view (∆LAd; 5.3 vs. 0.02 mm; *p* = 0.02).

Patients diagnosed with arterial hypertension were characterized by greater depression of LVEF during observation (∆ LVEF; − 7 vs. − 0.7%; *p* = 0.04).

The presence of diabetes mellitus was associated with greater elevation of *E*/*e*′ ratio (∆*E*/*e*′; 4.9 vs. 0.3; *p* = 0.002).

### Primary end point/cardiotoxicity

The primary end point occurred in ten patients (14.9%).

Patients with ≥ 10% decrease in LVEF in 12-month observation were characterized by higher initial blood glucose level (118.9 ± 37.6 vs. 92.2 ± 17.7 mg/dL, *p* = 0.03), elevated baseline heart rate (88.2 ± 14.5 vs. 77.3 ± 10.9, *p* = 0.01), higher frequency of coronary artery disease (30.0% vs. 6.25%, *p* = 0.04), dyslipidemia (60% vs. 15.6%, *p* = 0.02) and chronic kidney disease (25.0% vs. 3.3%, *p* = 0.04). BNP levels (373.4 vs. 355.0 pg/mL, *p* = 0.79) and troponin T levels (0.006 vs. 0.007 ng/mL, *p* = 0.68) were comparable in both groups.

The univariate analysis of different predictors of primary end point occurrence in the course of CTx is presented in Table 3. It was demonstrated that higher initial heart rate and the presence of dyslipidemia constitute statistically significant predictors of significant LVEF impairment in 12-month observation. There were no independent predictors of primary end point occurrence in the multivariate analysis.

**Table 3:** Univariate analysis of different predictors of ≥ 10% decrease in left ventricular ejection fraction within 12-month observation

Variable	OR	− 95% CL	+ 95% CL	*P*
Male	0.76	0.17	3.35	0.71
Non-DLBCL	1.70	0.37	7.85	0.48
Ann Arbor class III or IV	1.10	0.21	5.73	0.91
Specific treatment				
R-CHOP regimen	0.83	0.18	3.74	0.80
Anthracycline	2.16	0.21	22.02	0.51
Rituximab	0.81	0.16	4.12	0.80
Adjunctive treatment				
Beta blockers	0.75	0.12	4.53	0.75
ACEI	1.47	0.32	6.67	0.61
Diuretics	2.78	0.56	13.72	0.20
Anticoagulation	1.67	0.12	22.25	0.69
Baseline laboratory parameters				
RBC (per 1 mln/mm^3^)	0.33	0.06	1.87	0.20
WBC (per 1000/mm^3^)	0.89	0.63	1.26	0.51
Blood glucose (per 1 mg/dL)	1.04	0.99	1.09	0.08
TC (per 1 mg/dL)	0.99	0.92	1.05	0.63
LDL (per 1 mg/dL)	0.99	0.76	1.30	0.83
HDL (per 1 mg/dL)	0.89	0.03	23.88	0.74
Serum creatinine (per 1 mg/dL)	18.95	0.05	7656.06	0.33
eGFR < 60 mL/min	9.67	0.69	136.18	0.09
BNP	0.99	0.98	1.01	0.79
Troponin *T*	1.04	0.97	1.25	0.64
Comorbidities and cardiovascular risk factors				
HR (per 1/min)	1.09	1.01	1.17	0.03
SBP (per 1 mmHg)	0.99	0.95	1.05	0.93
DBP (per 1 mmHg)	0.98	0.91	1.05	0.55
Coronary artery disease	6.43	0.84	48.98	0.07
Arterial hypertension	2.5	0.56	11.19	0.22
Diabetes mellitus	1.75	0.25	12.04	0.56
Dyslipidemia	8.10	1.58	41.51	0.01
Cigarette smoking	1.46	0.34	6.36	0.60
Family history of cardiovascular disease	1.46	0.34	6.36	0.60
Baseline echocardiographic parameters				
LVEDD	0.97	0.88	1.08	0.61
LVESD	1.01	0.90	1.12	0.90
LVESV	0.94	0.87	1.01	0.08
LVEDV	0.99	0.96	1.01	0.27
SV	0.99	0.96	1.03	0.77
*E*/*e*’	1.26	0.96	1.67	0.10
RV mid-cavity diameter	0.77	0.59	1.0001	0.05
LA (LAX)	0.97	0.79	1.18	0.73
LAA (4Ch)	0.94	0.71	1.24	0.66
LA volume	0.96	0.89	1.03	0.23
Pericardial effusion	0.38	0.04	3.81	0.40
Mitral valve insufficiency	0.82	0.17	3.99	0.80

*BNP* B-type natriuretic peptide, *Non*-*DLBCL* non-diffuse large B cell lymphoma, *eGFR* estimated glomerular filtration rate, *TC* total cholesterol, *LDL* low-density lipoprotein, *HDL* high-density lipoprotein, *RBC* red blood cell count, *WBC* white blood cell count, *SBP*/*DBP* systolic and diastolic blood pressure, *HR* heart rate, *LVEF* left ventricular ejection fraction, *LVEDD*/*LVESD* left ventricular end-diastolic or end-systolic diameter, *LVEDV*/*LVESV* left ventricular end-diastolic or end-systolic volume, *RV* right ventricle, *LVSV* left ventricular stroke volume, *LA* (*LAX*) left atrial diameter in parasternal long axis view, *LAA* (*4Ch*) left atrial area in four-chamber view, *LA volume* left atrial volume calculated using Simpson method, *R*-*CHOP* cyclophosphamide, vincristine, prednisone, doxorubicin, rituximab

## Discussion

In the prospective multicenter ONCO-ECHO trial, we analyzed the echocardiographic indices in regard to clinical data in patients currently undergoing treatment for NHL. In a 12-month observation, the administration of CTx was found to be related to LV dilatation and impaired LV function. There was also an association between standard comorbidities and cardiovascular risk factors and negative LV remodeling. Asymptomatic cardiotoxicity of CTx was observed in 15% of patients. Although no fluctuations of the laboratory indices of cardiotoxicity, such as the troponin T concentration or BNP level, were observed, consistently increasing BNP levels were found in all of the NHL patients.

The management of NHL is based on standardized CTx regimens including some with well-documented cardiotoxicity. However, the NHL population is highly specific as it consists of elderly subjects with numerous comorbidities and pronounced cardiovascular risk factors. This is why clinical observation of echocardiographic indices in this clinical setting adds novel data.

In our population, five patients died during observation; however, the deaths were caused by the progression of NHL. No clinical manifestations of congestive heart failure or unplanned cardiovascular hospitalizations were found in the rest of the group. This suggests that CTx in NHL is relatively safe and did not lead to overt heart failure in a 12-month-long observation. On the other hand, we observed asymptomatic LV dilatation and impairment of the LV diastolic function.

CTx regimens which utilized anthracyclines have high efficacy in the treatment of solid tumors and hematological malignancies [2, 8, 11]; however, these regimens may induce negative cardiac side effects, i.e., negative LV remodeling [6]. A study by Cardinale et al. [17] corroborated an overall incidence of 9% of cardiotoxicity after anthracycline CTx, and 98% of cases occurred within the first year and were asymptomatic. The risk factors for anthracycline-induced cardiotoxicity involve: cumulative dose, infusion regimen and any condition that increases cardiac susceptibility, including preexisting cardiac disease, arterial hypertension, concomitant use of chemotherapy, as well as mediastinal radiation therapy and older age (> 65 years) [2, 4, 15]. It should be noted that anthracycline-related cardiotoxicity occurs to the greatest extent within the first year following initiation of treatment [17]. Thus, early detection and prompt therapy of cardiotoxicity seem crucial for substantial recovery of cardiac function.

According to the study protocol, cardiotoxicity was defined as *a* ≥ 10% decrease in the LVEF, which is in accordance with the currently available ESC recommendations [2]. In the study group, cardiotoxicity was reported in 15% of patients. Based on limited hitherto data on this topic, the cardiotoxicity of corresponding CTx regimens among elderly patients with DLBCL was broadly demonstrated [6, 13, 14]. Among patients receiving CTx for NHL, those who received doxorubicin were more likely to develop chronic heart failure [18]. In a study by Fridrik et al. [19] regarding CD20-positive DLBCL patients, the LVEF measurements were below 50% in 4.6% patients in the R-COMP arm and in 15.8% in the R-CHOP arm. On the other hand, in a historical retrospective analysis of patients with aggressive B cell non-Hodgkin’s lymphomas conducted between 1992 and 2012, the anthracycline-induced cardiomyopathy (AC-CMP) was observed in one-third of the enrolled patients [20].

Univariate analysis revealed that the presence of dyslipidemia and heart rate accurately predicted the occurrence of cardiotoxicity, while blood glucose level, reduced eGFR < 60 mL/min and coronary artery disease exhibited only a trend towards prediction of cardiac function impairment. Notwithstanding the fact that we did not find any independent predictors of cardiotoxicity, the results confirmed that high-risk subjects generally had a worse prognosis. This observation stays in accordance with other studies regarding coronary artery disease [20] and systemic arterial hypertension [21]. In the study by Hershman and al. [18], various cardiac risk factors increased risk of heart failure in NHL patients, but only hypertension was synergistic with anthracyclines. On the other hand, CTx may predispose individuals to the occurrence of both systemic hypertension and coronary artery disease [2, 11].

Modern approach to the management of anthracycline-induced cardiotoxicity in lymphoma patients is reflected by the study by Olivieri and coworkers [22]. In this report, the authors modified the CTx-related risk by replacing doxorubicin with non-pegylated liposomal doxorubicin and initiating the cardioprotective treatment immediately after subclinical cardiotoxicity was detected (echocardiography, troponin I level) [22]. This modification was applied to patients with cardiovascular risk factors and resulted in low incidence of cardiac complications [22].

The next advantage of the study was the assessment of the biomarkers. We analyzed the serum troponin T and BNP levels at consecutive time points of the observation. Contrary to the literature data suggesting prognostic value of Troponin I in the cardiac risk stratification of patients undergoing high-dose chemotherapy [17, 23], the troponin T levels remained stable and low. In all the NHL patients, the BNP levels were increased without any trend or relation to the point of the observation. Of note, the BNP levels exceeded the cutoff value characteristic for the diagnosis of heart failure (> 35 pg/mL) [24]. Similar data were published recently as the increased NT-proBNP levels were found in 67–90% of patients with CD20-positive DLBCL [19, 25]. In our study, none of the biomarkers had a predictive value for the occurrence of cardiotoxicity.

## Limitations of the study

We used *a* ≥ 10% decrease in the LVEF as a widely available cut-point to detect cardiotoxicity. Although TTE was performed by experienced echocardiographers with National Certification of experts in echocardiography fields, we did not have central echo laboratory and we did not analyze any interobserver variability. We are aware that there are more objective methods to assess LV systolic function available today, e.g., global longitudinal strain. Although assessing GLS to predict subsequent anthracycline-induced cardiotoxicity was described recently [26] and it is included in the ESC position paper on cancer treatments and cardiovascular toxicity [2], this method is not free from other limitations. The management of NHL was based on standardized CTx regimens; however, we are aware that the lack of data on cumulative doses of chemotherapeutic agents constitutes limitations of the study.

## Conclusions

The CTx of NHL, which primarily involves anthracyclines, may induce dilatation of the left ventricle, especially in patients with additional cardiovascular risk factors, including arterial hypertension, hyperlipidemia, diabetes mellitus and coronary artery disease.
